# The Tripartite Associations between Bacteriophage, *Wolbachia,* and Arthropods

**DOI:** 10.1371/journal.ppat.0020043

**Published:** 2006-05-19

**Authors:** Seth R Bordenstein, Michelle L Marshall, Adam J Fry, Ulandt Kim, Jennifer J Wernegreen

**Affiliations:** Josephine Bay Paul Center for Comparative Molecular Biology and Evolution, The Marine Biological Laboratory, Woods Hole, Massachusetts, United States of America; Stanford University, United States of America

## Abstract

By manipulating arthropod reproduction worldwide, the heritable endosymbiont *Wolbachia* has spread to pandemic levels. Little is known about the microbial basis of cytoplasmic incompatibility (CI) except that bacterial densities and percentages of infected sperm cysts associate with incompatibility strength. The recent discovery of a temperate bacteriophage (WO-B) of *Wolbachia* containing ankyrin-encoding genes and virulence factors has led to intensifying debate that bacteriophage WO-B induces CI. However, current hypotheses have not considered the separate roles that lytic and lysogenic phage might have on bacterial fitness and phenotype. Here we describe a set of quantitative approaches to characterize phage densities and its associations with bacterial densities and CI. We enumerated genome copy number of phage WO-B and *Wolbachia* and CI penetrance in supergroup A- and B-infected males of the parasitoid wasp Nasonia vitripennis. We report several findings: (1) variability in CI strength for A-infected males is positively associated with bacterial densities, as expected under the bacterial density model of CI, (2) phage and bacterial densities have a significant inverse association, as expected for an active lytic infection, and (3) CI strength and phage densities are inversely related in A-infected males; similarly, males expressing incomplete CI have significantly higher phage densities than males expressing complete CI. Ultrastructural analyses indicate that approximately 12% of the A *Wolbachia* have phage particles, and aggregations of these particles can putatively occur outside the *Wolbachia* cell. Physical interactions were observed between approximately 16% of the *Wolbachia* cells and spermatid tails. The results support a low to moderate frequency of lytic development in *Wolbachia* and an overall negative density relationship between bacteriophage and *Wolbachia*. The findings motivate a novel phage density model of CI in which lytic phage repress *Wolbachia* densities and therefore reproductive parasitism. We conclude that phage, *Wolbachia,* and arthropods form a tripartite symbiotic association in which all three are integral to understanding the biology of this widespread endosymbiosis. Clarifying the roles of lytic and lysogenic phage development in *Wolbachia* biology will effectively structure inquiries into this research topic.

## Synopsis

Symbiotic bacteria that are maternally inherited are widespread in terrestrial invertebrates. Such bacteria infect the cells of reproductive tissues and can have important evolutionary and developmental effects on the host. Often these inherited symbionts develop beneficial relationships with their hosts, but some species can also selfishly alter invertebrate reproduction to increase the numbers of infected females (the transmitting sex of the bacteria) in the population. Bacterial-mediated distortions such as male-killing, feminization, parthenogenesis induction, and cytoplasmic incompatibility are collectively known as “reproductive parasitism.” In this article, the investigators show that the associations between the most common reproductive parasite in the biosphere *(Wolbachia)* and a parasitic wasp host are affected by a mobile element—a temperate bacteriophage of *Wolbachia*. In contrast to recent reports that suggest bacteriophage WO-B may induce reproductive parasitism, the authors' quantitative and ultrastructural analyses indicate that lytic phage WO-B are lethal and therefore associate with a reduction in both *Wolbachia* densities and reproductive parasitism. Based on these data, the authors propose a phage density model in which lytic phage development specifically leads to a reduction, rather than induction, of reproductive parisitism. The study is among the first investigations to show that lytic bacteriophage inversely associate with the densities and phenotype of an obligate intracellular bacterium.

## Introduction

*Wolbachia* are α–proteobacterial endosymbionts that are recognized for their widespread distribution and inductions of reproductive parasitism, including feminization, male-killing, parthenogenesis, and cytoplasmic incompatibility (CI) in arthropods [1]. The success of these modifications has likely led to the worldwide spread of this maternally transmitted bacterium in at least 20% of all arthropod species [2–4]. Since insects comprise approximately 85% of all animal species, *Wolbachia* are by extrapolation one of the most abundant, obligate intracellular parasites in the biosphere and may affect major evolutionary processes including sexual selection [5], sex determination [6], and speciation [7–10] in their arthropod hosts. Like many maternally transmitted bacteria, *Wolbachia* typically infect the cells of the gonadal tissues.

CI is a common reproductive alteration induced by *Wolbachia* in insects. It is expressed most often as a one-way crossing incompatibility between infected males and uninfected females and imparts a relative fitness advantage to infected females by decreasing the fitness of uninfected females [11]. Detailed cytological studies in the haplodiploid wasp Nasonia vitripennis indicate that a sperm modification in infected males leads to delayed nuclear envelope breakdown of the male pronucleus [12] and resultant, improper condensation of the paternal chromosomes after fertilization of the uninfected egg [13]. Abnormal embryonic development of the egg ensues and the paternal chromosomes are ultimately lost. Maternal chromosomes segregate properly, resulting in haploid male progeny in N. vitripennis [14] but embryonic death in diploid insects like *Drosophila* [15]. When the fertilized egg is infected with the same *Wolbachia* strain as that in the male, fertilization and embryonic development occurs normally. There is considerable interest in the role of CI as a biocontrol tool to curb the spread of arthropod-borne pathogens and agricultural pests [16,17] as well as a rapid speciation mechanism in insects [10].

CI can be partial or complete, in which some or no progeny are produced in incompatible crosses between infected males and uninfected females, respectively [11]. Despite the efforts devoted to characterizing CI since the 1950′s, the microbial factors that shape CI variation have remained mostly elusive with the exception that *Wolbachia* densities positively associate with incompatibility levels within strains. This observation is known as the “bacterial density” model of CI [18] and numerous studies have confirmed the pattern with correlates between CI levels and counts of bacterial densities in infected eggs, infected sperm cysts, or whole adults [19–22]. Because *Wolbachia* are not present in sperm from infected males, the modification [23] leading to partial or complete CI must take place before the completion of spermatogenesis in infected testes [24–26]. Extrinsic factors that modulate incompatibility strength and type have been more readily detected and include host genetic background [27–29] and environmental stresses such as host age [15,30], heat treatment [31], mating history [32], and larval crowding [33] that can reduce bacterial densities. No studies have yet determined the effect of microbial factors, such as bacteriophage, on bacterial density variation and the expression of CI. Furthermore, the general function of bacteriophages in maternally transmitted symbionts is paradoxical itself since these bacteria tend to have a confined intracellular niche and an accelerated rate of mobile element loss [34–36].

*Wolbachia,* Candidatus *Hamiltonella defensa,* and *Spiroplasma* are the only known maternally transmitted arthropod endosymbionts with bacteriophage elements [37–39]. The full genome sequence of Wolbachia wMel revealed three prophage regions: a small pyocin-like element, *w*Mel WO-A, and *w*Mel WO-B [40]. Recently, bacteriophage WO-B of *Wolbachia* has received heightened interest in regards to its ability to form mature virion particles [41,42], horizontally transfer between coinfecting strains [43,44], and serve as a molecular marker for *Wolbachia* strain typing [45]. Ankyrin repeats—a common protein sequence motif that mediate interactions between a wide spectrum of proteins including cytoskeletal organizers and cell cycle regulators—are embedded in genes of prophage WO-A and WO-B that are predicted to be highly expressed [40]. Notably, CI appears to result from changes in cell cycle regulatory proteins [46] that might be mediated by genes containing such ankyrin repeats. In addition, the 20.5-kb WO-B virion genome also carries a gene with putative virulence function *(VrlC)* that may encode effector proteins associated with the phage or type IV secretion system of *Wolbachia* [42]. Sex-specific expression of two phage-associated genes has also been observed [29,47]. For these reasons, bacteriophage WO-B has been tentatively proposed as a genetic candidate for inducing CI [29,42,48], but there is inconsistent evidence on whether WO-B sequence diversity correlates with CI crossing type [49,50]. It remains unclear whether bacteriophage actually encode genes directly involved in CI, and whether the temperate WO-B can influence *Wolbachia* during its lytic or lysogenic development. We suggest that clarifying the separate roles of lytic and lysogenic phage development in *Wolbachia* biology will effectively structure inquiries into this research topic.

Here we consider the role of lytic development in association with *Wolbachia* densities and penetrance of reproductive parasitism. We propose a “phage density” model in which lytic development of temperate bacteriophage WO-B, encompassing processes such as DNA replication, virion formation, and cell lysis, leads to the reduction rather than induction of CI (Figure 1). Our rational is that lysis or negative physiological effects of replicating phage on *Wolbachia* cell cycle processes could reduce or maintain low bacterial densities. Since *Wolbachia* densities positively associate with incompatibility levels in many systems [19–22], phage replication could lead to a reduction in CI. This hypothesis is simple and clarifies the role that lytic phage may have on the biology of *Wolbachia,* but does not rule out a role for lysogenic phage in the expression of CI. Revealing the microbial genetic factors that modulate CI will significantly enhance our understanding of how *Wolbachia* endosymbionts override normal host reproductive strategies. In addition, the studies described here comprise the first investigation of how bacteriophage lifecycle generally associates with the density and phenotype of an obligate intracellular bacterium.

**Figure 1:**
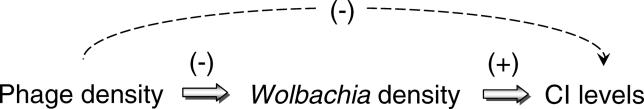
A Phage Density Model of CI This flowchart depicts the predicted associations, i.e., (−) negative and (+) positive, between phage densities, *Wolbachia* densities, and CI levels with arrows connecting the associated variables. The flowchart is based on the following hypothesis: if lytic development of temperate phage WO-B leads to bacterial lysis or slowed cell divisions, the relative copy number of phages per bacterium may negatively associate with the relative copy number of *Wolbachia* per host, which in turn is well established to positively associate with CI levels. Under this scenario, phage densities can have a secondary negative association with CI levels.

## Results/Discussion

We report a set of interconnected experiments that highlight the density and ultrastructure relationships between bacteriophage WO-B, *Wolbachia,* and by association, reproductive parasitism. By enumerating CI levels and microbial abundances from individual A- and B-infected N. vitripennis males, we characterize variation in CI strength and phage and bacterial densities, and analyze the correlates between these three variables. Ultrastructural analyses are conducted to validate the quantitative PCR findings and revealed a moderate frequency of virion-containing *Wolbachia* in an often degenerate and lysed state, as well as putative aggregations of extracellular virions. Observed interactions between *Wolbachia* membrane and individualized spermatids are also reported.

### CI in N. vitripennis

In the haplodiploid *N. vitripennis,* compatibility is measured by the number of diploid females produced among progeny because only diploid females result from fertilization. Since normal compatible sex ratios under the experimental design are female biased (80% to 95%), incompatibility is expressed as a reduction in the number of female progeny.

To measure *Wolbachia*-induced CI, we first crossed single infected males of N. vitripennis harboring one of the two main strains of arthropod *Wolbachia* (A or B) to single uninfected females and scored F1 progeny numbers and sex ratios from these incompatible crosses (Table 1). Control crosses with uninfected males and females were run concurrently for comparison. Crosses with A-infected and B-infected males elicit significantly different patterns of CI. B-infected males induce complete incompatibility as evident by a 100% reduction in the number of diploid females in comparison to that of the uninfected (U) control crosses (Mann-Whitney *U, p* < 0.0001), while A-infected males induce significant, partial incompatibility (67% reduction) in comparison to uninfecteds (Mann-Whitney *U, p* < 0.0001) (Figure 2). Because N. vitripennis harbor putative nuclear genes that convert fertilized eggs from CI crosses into haploid eggs carrying only the maternal chromosomes [51], there is also a corresponding increase in the number of haploid males in both sets of incompatible crosses compared to that of the control crosses (Mann-Whitney *U, p* < 0.0001). Effects of host genetic background on the CI level differences observed between A and B *Wolbachia* are mitigated by the derivation of these stocks from the same doubly-infected strain [52] and the consistency of our CI penetrance data with previous findings using these same N. vitripennis stocks [51,52].

**Table 1:**
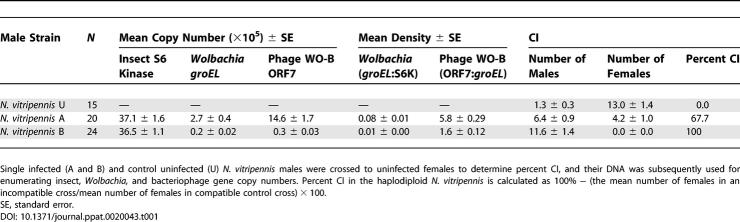
Summary Statistics from Quantitative PCR and CI Experiments Using A and B *Wolbachia*

**Figure 2:**
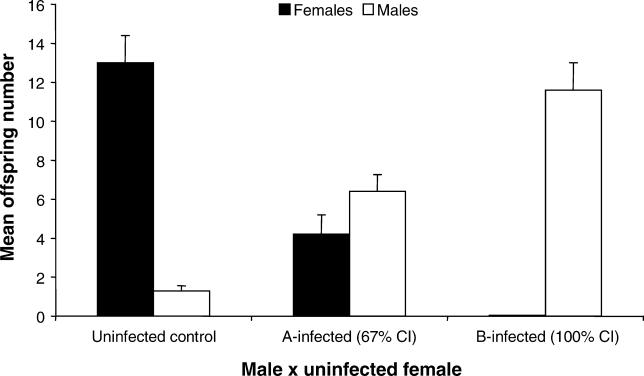
*Wolbachia*-Induced CI in N. vitripennis Mean offspring number ± SE are shown for crosses between uninfected females and single males that are uninfected (*n* = 15), A-infected (*n* = 20), or B-infected (*n* = 24). % CI is calculated as 100% − (mean number of females in an incompatible cross/mean number of females in the control compatible cross) × 100.

### Relationship of Phage WO-B and *Wolbachia* Copy Number

To examine *in vivo* copy numbers of bacteriophage and *Wolbachia* from the infected males above, we used real-time quantitative polymerase chain reaction (RT-qPCR) and enumerated single copy genes of the N. vitripennis genome (S6 kinase or *S6K*), *Wolbachia* genome *(groEL),* and phage WO-B genome (ORF7) (Table 1). These gene copy counts then reflect total microbial abundances in N. vitripennis males. The plaque assay is the traditional method for the quantification of bacteriophages, but is not applicable to unculturable endosymbionts. RT-qPCR is instead a more precise and reliable alternative to the plaque assay [53] and allows simultaneous quantification of different gene copy numbers using a common methodology.

Figure 3 shows that total bacteriophage WO-B abundance is strongly correlated with total bacterial abundance for both A *Wolbachia* (rho = 0.9814, *p* < 0.0001) and B *Wolbachia* (rho = 0.9537, *p* < 0.0001), as expected for a temperate bacteriophage in which a lysogenic phage is cotransmitted with the bacterial host and lytic infection depends on the bacterial encounter frequency. However, the total phage and bacterial gene copy numbers are strikingly far greater for A *Wolbachia* than B *Wolbachia* (Table 1). The bacteriophage ORF7 and *Wolbachia groEL* copy number means for A-infected males are at least an order of magnitude greater than that of B-infected males, respectively (Mann-Whitney *U*, *p* < 0.0001 for both comparisons). The larger genomic abundances of A versus B *Wolbachia* and their associated phage are noteworthy given that A *Wolbachia*–infected males express incomplete CI while B *Wolbachia*–infected males express complete CI. A *Wolbachia* apparently have a higher density threshold for inducing complete CI than B *Wolbachia* in N. vitripennis males. These total abundance differences are not due to host body size differences between the A- and B-infected males (Insect S6K counts, Mann-Whitney *U, p* = 0.8137) and indicate, as other studies have, that abundance data among divergent *Wolbachia* strains are not comparable in relation to CI level variability [54,55].

**Figure 3:**
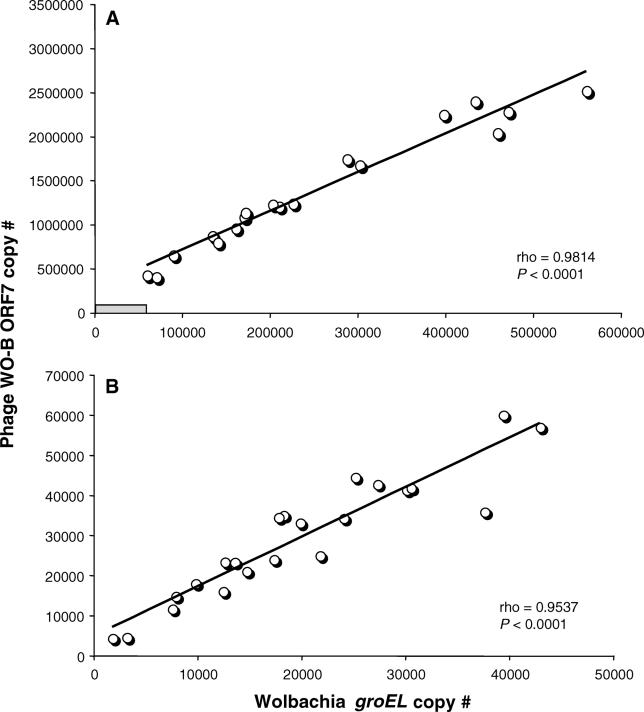
The Associations of Temperate Phage WO-B and *Wolbachia* Abundance Points on the charts denote the absolute copy numbers of a single-copy gene for phage WO-B and *Wolbachia* in individual adults of (A) A-infected males and (B) B-infected males of N. vitripennis. Correlation coefficients and significances are calculated according to the nonparametric method of Spearman's rho. In (A), the gray rectangle denotes the relative range of copy number variation for B-infected males, as depicted in larger scale in (B).

### Associations between Phage, Bacteria, and CI Penetrance

We tested the three associations predicted by a phage density model (Figure 1). We used data strictly from the A *Wolbachia*–infected males as they show variability in the strength of incompatibility. A-infected males inducing nearly complete CI (specified as none to one F1 female) have an average *Wolbachia* density (*groEL* copy number*/S6K* copy number) of 0.114, while B-infected males which all induce complete CI have an average density of 0.01 (*p* < 0.0001, Mann-Whitney *U*). Furthermore, A-infected males inducing partial CI (specified as two F1 females or more) have a significantly lower average density (0.048) than similarly infected males expressing complete CI (*p* = 0.0036, Mann-Whitney *U*). The results are again consistent with the bacterial density model and an inability to compare density relationships of the divergent A and B *Wolbachia*.

Calculations of phage copies per *Wolbachia* copies support the temperate lifestyle of WO-B in N. vitripennis A *Wolbachia*. We previously identified four different ORF7 paralogs in the N. vitripennis A *Wolbachia* through cloning and sequencing of heterogeneous PCR products [44]. Thus, at a minimum, we expect a ratio of four phage genes per bacterium during lysogeny. Our RT-qPCR data confirm this estimate, as the phage-to-bacteria ratio exceeds 4 in all cases (Figure 4A). The phage WO-B density (ORF7 copy number/*groEL* copy number) ranges from 4.44 to 7.31 with an average of 5.83. We also previously identified one ORF7 homolog in the B *Wolbachia* and the enumerated phage-to-bacteria ratio exceeds 1 in all cases (unpublished data) with an average of 1.6 (Table 1). Genomic copy number per cell in *Wolbachia* of Brugia malayi was estimated to be one [56]. If this holds in the A *Wolbachia* of *N. vitripennis,* then our results may be interpreted as an average ratio of 5.83 phages per bacterium. The consistency between the sequence data and quantitative PCR data suggests that phage WO-B may encode repressors that prevent lysogeny of genetically identical or similar phage. Such repression is typical of other temperate bacteriophages. The overall variation in phage densities suggests that phage are replicating and undergoing lytic development at a moderate rate. Quantitative electron microscopy data support this inference (see below).

**Figure 4:**
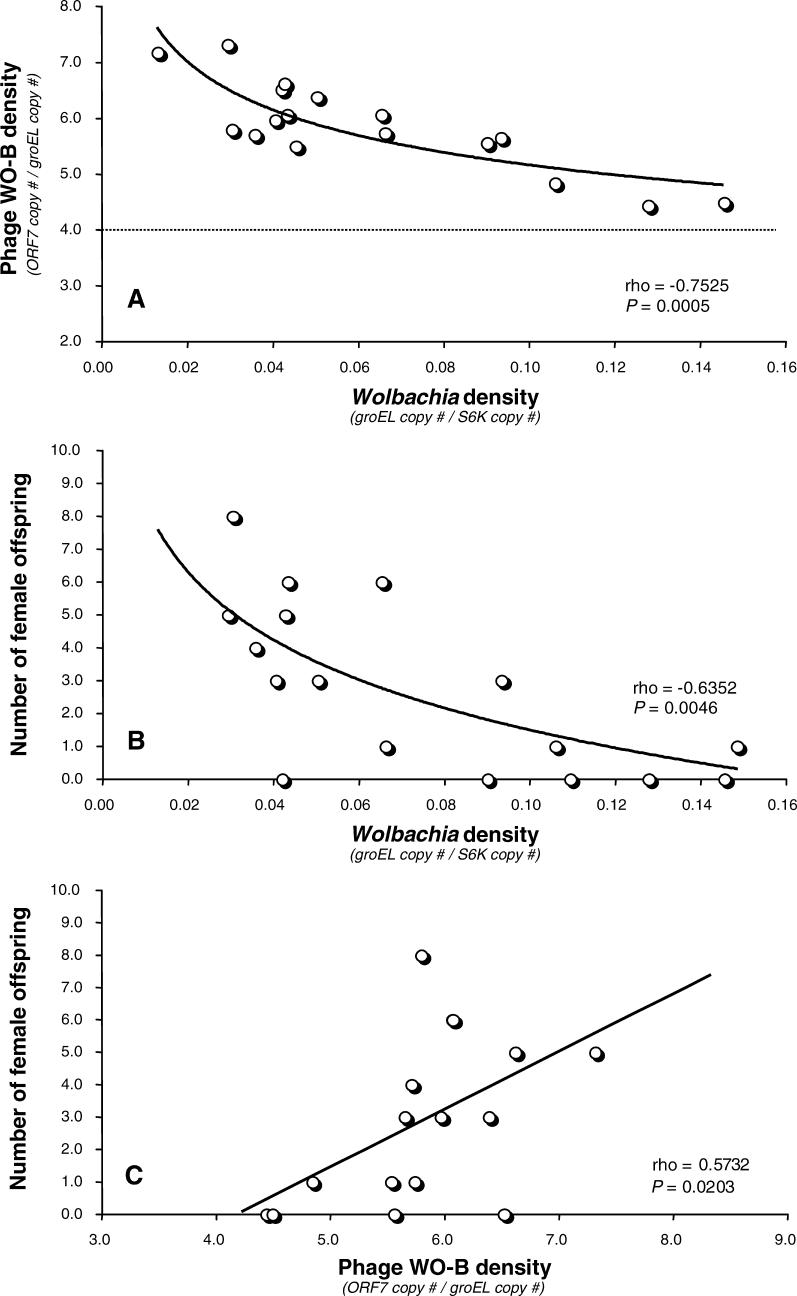
The Relationships between Phage Density, *Wolbachia* Density, and CI Level Points on the charts denote the relative phage WO-B density, relative *Wolbachia* density, and the number of female (diploid) offspring produced from cytoplasmically incompatible crosses using A-infected males. CI in haplodiploid species is expressed as a reduced number of female offspring. In (A), the dashed line denotes the estimated number of different lysogenic WO-B prophages per A-*Wolbachia* genome as determined by OFR7 amplification, cloning, and sequencing of heterogeneous copies [44].

The phage density model in Figure 1 directly predicts that phage densities will inversely associate with *Wolbachia* densities based on the basic tenets that (i) bacteriophages are predators of bacteria and (ii) lytic development will inhibit bacterial replication or induce cell lysis. Microscopic observations of virion-containing *Wolbachia* exhibit cell lysis [41] or intracellular pyknotic-like patches indicative of cell death [37]. Consistent with these observations, we report a strong and significant inverse association between phage and A *Wolbachia* densities in N. vitripennis males (rho = −0.7525, *p* = 0.0005, Figure 4A). For instance, the highest WO-B densities (up to 7.31 per *Wolbachia* genome) have the lowest *Wolbachia* densities (Figure 4A) and lowest absolute abundance of *Wolbachia* (less than 1 × 10^5^ *groEL* copies) for the entire dataset and vice versa. These findings specify that samples with the highest densities of lytic phage WO-B associate with *Wolbachia* experiencing the slowest rates of replication or a phage-mediated reduction in *Wolbachia* densities.

The RT-qPCR data also validate the bacterial density model: a direct, positive relationship between *Wolbachia* densities and CI strength (Figure 4B). Since CI in haplodiploids is measured as a reduction in the number of diploid females produced, we present the analysis as number of surviving, female offspring produced from incompatible crosses plotted against microbial densities. As a result, the predicted trend is that low numbers of female offspring, i.e., high CI inducers, will associate with high *Wolbachia* densities. As predicted, we found that female offspring numbers and *Wolbachia* densities in infected males showed a significant negative association (rho = −0.6352, *p* = 0.0046). The higher numbers of female offspring per male (more compatibility) associate with the lower *Wolbachia* densities, while the lower numbers of female offspring per male (more incompatibility) associate with the higher *Wolbachia* densities. The data therefore corroborate the bacterial density model in infected N. vitripennis males, in comparison to previous techniques that scored bacterial densities in eggs [18].

From the negative association between phage WO-B and *Wolbachia* densities, and the positive association between *Wolbachia* densities and CI levels, it follows that phage WO-B densities could indirectly associate with CI penetrance by virtue of a second-order correlation (Figure 1). The basic reason is that phage-mediated cell lysis will simultaneously decrease *Wolbachia* densities and increase phage densities, as evident in Figure 4A. Percentages of lytic *Wolbachia* per male are the ideal data point to correlate to CI levels, but are difficult to assess in practice owing to the unculturability of *Wolbachia*. Enumerated phage densities are thus a proxy for lytic development since increasing phage densities results from lytic development. Caveats to using this proxy include (i) variation in phage densities could be accounted for by variability in the number of virions produced per bacteria. However, if the variation was strictly due to variable numbers of virions produced by the same number of *Wolbachia* in infected males, then we would not observe the corresponding variation in A *Wolbachia* abundances evident from the correlate in Figure 3A and (ii) *Wolbachia* densities do not perfectly correlate with phage densities or CI penetrance as evident by the coefficient values of rho = −0.7525 (Figure 4A) and rho = −0.6352 (Figure 4B), respectively. The variation not accounted for in these correlation coefficients will further amplify the noise in the indirect, second-order correlation analysis between phage densities and CI levels.

Despite the caveats, Spearman's rho correlation analysis reveals a positive and tentatively significant relationship between phage WO-B densities and female offspring numbers following the removal of four outliers identified by the jackknifed Mahalanobis Distance (rho = 0.5732, *p* = 0.0203, Figure 4C). The higher numbers of female offspring per male (more compatibility) associate with the higher phage WO-B densities (more lytic development), while the lower numbers of female offspring per male (more incompatibility) associate with the lower phage WO-B densities (less lytic development). The data thus tentatively corroborate the phage density model in infected N. vitripennis males. As a further analysis of the data, we performed two nonparametric pairwise comparisons to test the hypothesis that males expressing nearly complete CI (none to one F1 female) have lower phage densities than males expressing incomplete CI (two F1 females or more). The Mann-Whitney *U* tests are significant both before (*p* = 0.0261) and after (*p* = 0.0064) the removal of the four outliers identified above.

In total, the combination of support for all three associations is consistent with an inverse relationship between lytic phage and *Wolbachia* densities and reproductive parasitism. Based on these tripartite associations, we suggest that lytic phage indirectly repress CI by negatively affecting *Wolbachia* fitness through cell lysis and/or slowed replication rates. However, we caution that this correlative evidence does not prove causation and the role of lysogenic phage (i.e., prophage gene expression) in reproductive parasitism awaits a full transcriptome characterization.

### Confirmation of Lytic Development of Bacteriophage WO-B

We employed transmission electron microscopy (TEM) to directly confirm the lytic phage development evident from the quantitative PCR studies. Figure 5A shows an electron micrograph of negatively stained virion particles collected from whole A-infected *Nasonia* homogenizations and serial filtrations through 0.45-μm and 0.22-μm filters. Genomic isolation of these filtrated virions and PCR assays indicated that the filtrate contains phage WO-B, and is free of *Wolbachia* contamination. The virion sizes are the same as those observed inside *Wolbachia* from micrographs of random thin sections of infected, pupal testes (Figure 5B–5E). These thin section micrographs revealed a range of virion-containing (*n* = 6) and virion-free *Wolbachia* (*n* = 45). *Wolbachia* bacteria ranged in size from 300 nm to 1 μm in length and 250 to 590 nm in width, with usually three observable membranes consisting of the host vacuole, the cell wall, and plasma membrane. Spermatogenesis is synchronized in N. vitripennis and mostly complete during the late pupal stages [57]. As a result, *Wolbachia* were closely associated with the sperm flagellar axoneme and mitochondrial derivatives of individualized spermatids (Figure 5B), which is consistent with other insects [58,59]. Based on the observed proportion of virion-containing *Wolbachia* in infected testes (Table 2, Figure 5B–5E), 11.76% of *Wolbachia* cells were determined to be experiencing lytic phage development. This percentage is approximately less than half of that observed in micrographs of ovary-infecting *Wolbachia* in Culex pipiens [37]. The mean number of virions per infected *Wolbachia* in our observations was 24.7. Based on these estimates and sequence data indicating four lysogenic phages per *Wolbachia* [44], we can back-calculate a tentative estimate of the TEM-observed ratio of phage copies per *Wolbachia* using the following equation: [(the average number of observed virions per *Wolbachia* plus an estimated four lysogenic phages per *Wolbachia*) × (observed frequency of virion-containing *Wolbachia*)] + [(four lysogenic phages per *Wolbachia*) × (the observed frequency of virion-free *Wolbachia*)]. This equation [(24.66 + 4.0)(0.1176)] + [(4.0)(0.8829)] yields an estimate of 6.9 phage copies per *Wolbachia,* a number that parallels the real-time quantitative PCR estimate of an average density of 5.8 phages per bacterium. Despite many assumptions requiring proper caution, it can be concluded that the consistency of the RT-qPCR and TEM studies, and relatively low frequency of observed virion-containing *Wolbachia* (11.78%), produces an overall, moderate intrahost ratio of phage to bacteria.

**Figure 5:**
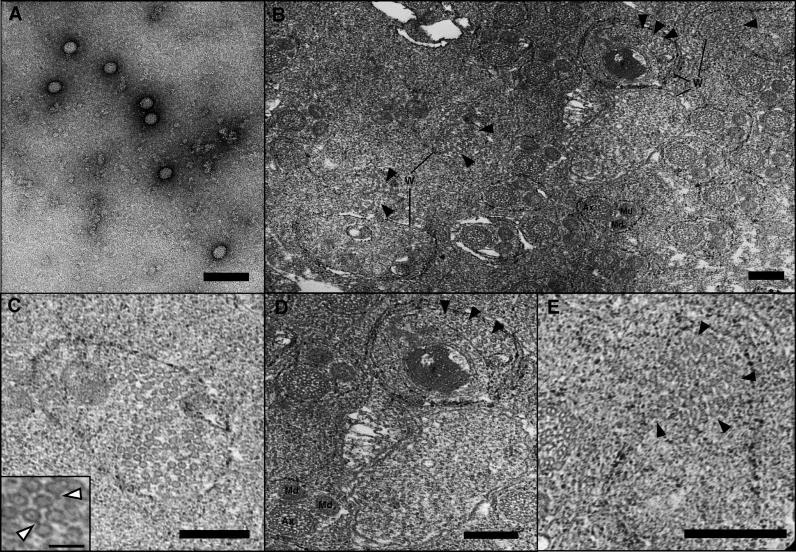
*Wolbachia* Infecting the Testes of N. vitripennis (A) Negatively stained virus particles of uniform size from PEG-precipitated preparations of A-infected N. vitripennis adults. No tail-like structures were apparent, perhaps due to disruption during the purification process. Bar = 100 nm. (B) Low-resolution transmission electron micrograph of individualized spermatids and *Wolbachia* in N. vitripennis pupal testes. Ax and Md denote spermatid axonemes and mitochondrial derivatives. Several of the noted *Wolbachia* cells (W) are shown in higher resolution in Figure 5C–5E. Solid arrowheads denote phage particles. Bar = 200 nm. (C) A high density of phage particles within *Wolbachia* is shown; phage tails are occasionally visible and noted by white arrowheads in the inset. Bar = 200 nm; inset bar = 100 nm. (D) Virion-free (lower right) and virion-containing *Wolbachia* (upper right) localized near two N. vitripennis spermatids are shown. Solid arrowheads denote phage particles inside *Wolbachia*, and Ax and Md denote spermatid axonemes and mitochondrial derivatives, respectively. Bar = 200 nm. (E) Solid arrowheads denote phage particles dispersing from within putatively lysed *Wolbachia* into the extracellular matrix. Bar = 200 nm.

**Table 2:**
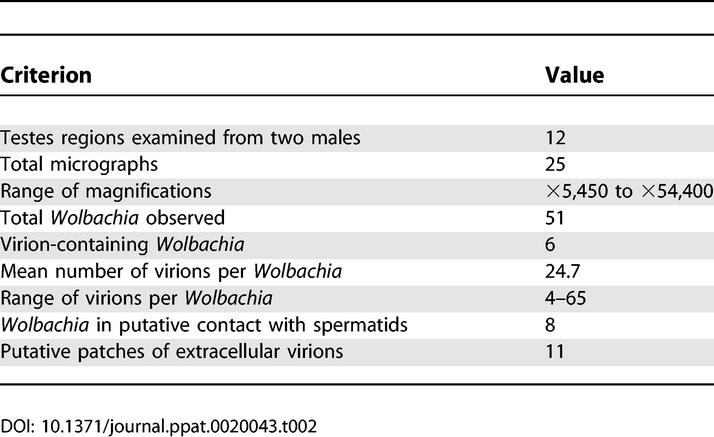
Summary of TEM in Late Pupal Testes

The icosehedral virions of *Wolbachia* ranged in diameter from 35 to 45 nm, some of which had an observable tail-like structure that ranged in length from 10 to 20 nm. Bacteriophage tails are typically required for host membrane attachment and infection. Particles were distributed along the inner membrane and in the middle of the cell, which was often irregularly shaped, degenerative, and lysed. We observed virion-containing *Wolbachia* with dense masses in the middle of the cell and detached membrane structures analogous to phage-infected *Chlamydia* [60] (Figure 5D). The range of virions per *Wolbachia* widely varied from four to 65. In at least three of six cases, virions were observed leaving lysed cells into the extracellular matrix (Figure 5E), indicating the phage particles are lethal to *Wolbachia*. Aggregations of similarly sized viral particles were also observed in the extracellular matrix of the testes (Figure 6A–6C). No detectable *Wolbachia* occurred around these patches, indicating that these virion clusters probably departed from the bacterial cell. Single membrane structures completely or partially surrounding the patches may be present, but can not be confirmed based on the micrographs.

**Figure 6:**
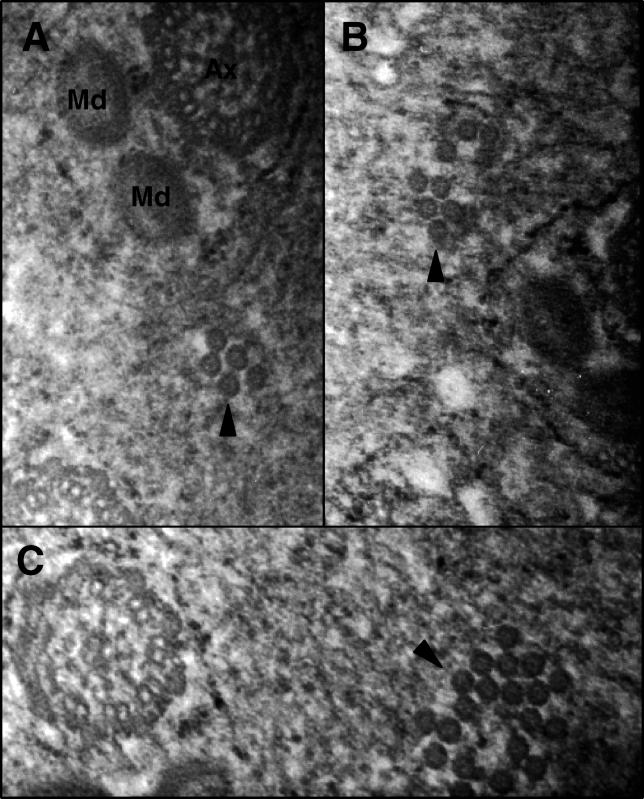
Aggregations of Putative Extracellular Virions Solid arrowheads denote phage particles with no detectable *Wolbachia* membrane surrounding them. In some cases, a single membrane structure can be seen either completely or partially surrounding the phage particle patches. (A) A patch of five virions near an individualized spermatid, for size reference. (B) A patch of nine virions to the left of an individualized spermatid. (C) A patch of 20 virions near a flagellar axoneme with potentially a single membrane structure surrounding the patch.

### Interactions between *Wolbachia* Cells and Spermatids

It has been hypothesized that the mechanism of CI in crosses between infected males and uninfected females involves an imprinting effect in the testes since mature sperm do not harbor *Wolbachia* [11,23]. Whether sperm imprinting is due to *Wolbachia*-secreted proteins or the uptake of host proteins by *Wolbachia* is currently unresolved, but the discovery of a type IV secretion system in *Wolbachia* has heightened interest in the function of secreted proteins [61]. Our observations with TEM of infected N. vitripennis testes unexpectedly revealed cases of *Wolbachia* membrane in contact with or surrounding N. vitripennis spermatids (Figure 7). *Wolbachia* are typically spherical or rod-shaped, but *Wolbachia* also appeared twisted in the direction of contact with multiple spermatids (Figure 7A). Only virion-free *Wolbachia* were observed to contact the spermatids. Of the total observed *Wolbachia,* 15.68% were observed in putative contact with membranes of spermatid tails. These observations present the possibility that *Wolbachia* may be responsible for expressing a sperm imprinting effect through direct physical interactions with the developing spermatids, or alternatively in the general tissue matrix of the testes. If the mechanism of CI is dependent or influenced by *Wolbachia*-spermatid contact, then variation in incompatibility strength could be determined by the proportion of spermatids contacted by *Wolbachia*. No direct evidence currently supports this. Recent TEM observations in infected Drosophila simulans testes indicated that *Wolbachia* reach their highest densities around elongating spermatids [59]. These observations and mechanistic hypotheses warrant future microscopy studies to determine how common the physical associations are in other incompatibility-inducing *Wolbachia*. Without further investigations, it is not clear what functional relationship, if any, this contact may actually have. We believe these photographs comprise the first documentation of physical contacts between *Wolbachia* and individualized spermatids, probably due to a preference for electron microscopy work in the transmitting sex-infected females.

**Figure 7:**
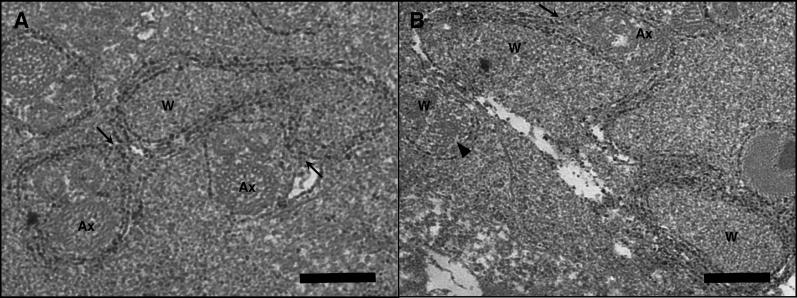
*Wolbachia* Membrane Associates with Spermatid Tails in the Testes of N. vitripennis Arrows indicate regions of contact between virion-free *Wolbachia* and spermatids. Ax denotes flagellar axonemes. W denotes *Wolbachia*. (A) Contact between a single *Wolbachia* cell and two individual spermatids. Bar = 200 nm. (B) The membrane of a dividing *Wolbachia* (upper left) associates with a putative spermatid along the junction indicated by arrow. Ax denotes axonemal structure. Solid arrowhead denotes virion particles within neighboring *Wolbachia* (middle left). Bar = 200 nm.

### Summary

Symbiosis impacts all levels of organic complexity. Bacterial endosymbiosis in particular is a hallmark example of the intimate, binary interactions between prokaryotes and eukaryotes. Such interactions are common in animals and have facilitated important insights into the processes affecting cellular complexity, bacterial genome size, and organelle evolution. The surprising discoveries of bacteriophage in some of these endosymbiotic bacteria holds enormous value for further understanding these symbiotic associations and the development of genetic tools in these unculturable systems.

In this study, we examined the tripartite associations and ultrastructure of a phage, bacterial endosymbiont, and host arthropod that collectively represent a widespread and evolutionary important symbiotic system. The effects that lytic phage growth and virion production can have on this tripartite symbiosis span important evolutionary and ecological processes such as lateral phage transfer between *Wolbachia* (virion production is a requisite for mobility and transduction), potential modulation of intracellular *Wolbachia* densities (lytic development may repress bacterial densities), and, by association, penetrance of *Wolbachia*-mediated reproductive parasitism such as CI.

Our quantitative and ultrastructural analyses comprise the first studies to illustrate a negative density relationship between phage WO-B and *Wolbachia,* as expected for an active lytic phage. Ultrastructural analyses confirm the ability of *Wolbachia* phage to form virion particles and lyse the host cell. Furthermore, the negative association between CI levels and phage densities suggests that the genes encoding CI “toxins” apparently are not expressed in replicating or mature phage. Otherwise, we would expect a positive correlation between phage densities and CI levels. Genes in phage WO-B may still encode CI “toxins” while the genome is integrated as a prophage during lysogeny, but a complete transcriptome characterization of the phage during lysogeny is necessary to address this issue. Sex-specific expression of phage-related genes is consistent with a possible role of lysogenic phage in reproductive parasitism and has been observed in infected *Culex* mosquitoes [29,47]. However, an alternative explanation for sex-specific expression is that bacteriophage may have different rates of lytic development in males and females. Selection for increased bacterial transmission efficiency at the bacterial level or suppression of incompatibility levels at the host level may for example promote the evolution of higher lytic development rates and thus expression in infected males. Either of these hypotheses are consistent with a phage density model in which phage are strictly parasitic to *Wolbachia* and do not impart a benefit to the bacterium.

One hypothesis for the widespread distribution of phage WO-B [44] based on this speculation is that the host, rather than *Wolbachia,* benefits from the presence of a lytic phage since host level and phage level selection could both favor an active bacteriophage infection that lyses *Wolbachia* cells and thus reduces the bacterial load in the arthropod host. This host level selection could also explain its absence from mutualistic *Wolbachia* in filarial nematodes [62] in which host level selection would select against a lytic phage of a mutualistic *Wolbachia* that is required for host oogenesis and larval development. The results presented here also raise the interesting possibility that common environmental (e.g., temperature) or host genetic factors known to modulate *Wolbachia* densities do so by inducing prophage excision, virion production, and cell lysis. Future investigations should simultaneously examine the effects of these extrinsic factors on phage and insect endosymbiont densities. Finally, an active and inducible temperate phage in *Wolbachia* is a promising genetic tool to engineer *Wolbachia* for biocontrol strategies or as a biotherapy tool to combat the devastating pathologies of *Wolbachia*-assisted filariasis in humans. It is clear that the *Wolbachia* symbiosis has been shaped by at least three genetic entities and experimental and theoretical studies on their interactions will collectively assemble a general model of the host-reproductive parasite interactions.

## Materials and Methods

### Insect strains.

N. vitripennis are gregarious parasitoid wasps of fly pupae. They are raised on Sarcophaga bullata (flesh fly) pupae in the laboratory, with constant light and temperature (21 °C). Three strains were used in these studies: a single A-infected strain (12.1), a single B-infected lab strain (4.9), and an uninfected strain (13.2). These strains were derived in 1996 from a double AB-infected isofemale strain by spontaneous loss of *Wolbachia* following prolonged diapause [52].

### Tests of CI.

All crosses were set up as single pair matings between uninfected virgin females and virgin males of each of the three strains. Only those pairings where copulation occurred within 10 min of observation were used. Males were immediately frozen at −80 °C, and each female was provided with four hosts for feeding and egg laying. After 48 h, the females were transferred to new vials and given a single host for 6 h. Females were then discarded from each vial and the parasitized hosts were left undisturbed until adult emergence in approximately 2 wk. Adults were scored upon death for sex ratios and total family size. Two replicate experiments were performed 8 mo apart.

### RT-qPCR.

Genomic DNA was isolated using the DNeasy Tissue Kit (Qiagen, Valencia, California, United States) from single adult males and suspended in 70 μl of double-distilled sterile water. Infection status on a subset of these adults was confirmed by PCR with *Wolbachia*-specific primers. RT-qPCR was performed in an iCycler system (Bio-Rad, Hercules, California, United States). Reaction volumes of 25 μl contained 12.5 μl of BioRad SYBR Green Supermix, 10.5 μl of sterile water, 0.5 μl each of 10 μM forward and reverse primer, and 1 μl of target DNA in single wells of a 96-well plate (Bio-Rad). For the selective amplification of a small portion of the *Nasonia S6 Kinase* gene (133 bp), *Wolbachia groEL* (97 bp), and WO-B ORF7 (125 bp), the following primers were designed and employed: NvS6KQTF2 (5′-GGCATTATCTACAGAGATTTG-3′), NvS6KQTR2 (5′-GCTATATGACCTTCTGTATCAAG-3′), NvWGroQTF1 (5′-CAACCTTTACTTCCTATTCTTG-3′), NvWGroQTR1 (5′-CTAAAGTGCTTAATGCTTCACCTTC-3′), NvWOF1 (5′-GTCTGGAAAGCTTACAAAAAG-3′), and NvWOR1 (5′-CTCGCCAAAATATAGCCCTGC-3′). PCR conditions comprised an initial melting at 95 °C for 3 min, followed by 40 cycles with melting at 95 °C (15 s) and primer annealing at 55.6 °C (1 min). A melting curve analysis was performed at the end of the PCRs to check for primer-dimers and nonspecific amplification. Standard curves for each gene were constructed with a log10 dilution series of known amounts of PCR products cloned into plasmid vectors using the TOPO TA cloning kit (Invitrogen, Carlsbad, California, United States). RT-qPCR assays were performed in triplicate on all DNAs and two replicate experiments were performed in total.

### Statistics.

Correlation coefficients were calculated using the nonparametric Spearman's rho methods in JMP version 5.0.1a (SAS Institute Inc., Cary, North Carolina, United States) Multivariate outliers were identified by the jackknifed Mahalanobis Distance in JMP and removed from the datasets (two to four of approximately 24 total data points per dataset). Outliers can artificially increase or decrease the value of a correlation coefficient and their removal can have recommended benefits that are not typically considered in practice [63,64]. Removals of the outliers did not affect the significance of rho for all analyses except that in Figure 4C in which rho = 0.3339 and *p* = 0.15 prior to removal of four outliers. We expect more variability in this correlation analysis since the phage densities and CI strength are only indirectly related. Linear or logarithmic trend lines were added to the charts to illustrate patterns using Microsoft Excel.

Small plate effects are common in RT-qPCR experiments and were apparent by a trend of slightly elevated or reduced threshold cycle (Ct) values for the same template DNA used for the standard curve across two different plates. Before normalization of the plate effects, individual correlation coefficients (Spearman's rho) on the data from each plate were determined to specify the same association trends (positive or negative) by a homogeneity test ([65], Box 15.4) using the z*-transformation of data for small datasets. Normalization was then performed by reconstructing the RT-qPCR standard curve with the average threshold cycles of the enumerated dilution series across separate plates. Ct values for experimental DNAs for each gene were compared against the “average” standard curve to specify the normalized starting quantities of template DNA. No plate effects were observed after data normalization.

### Electron microscopy.

Whole-insect homogenates from infected N. vitripennis adults (0.5 g) were isolated using buffers and centrifugation steps previously described [42], with the exception that particles were serially filtrated through 0.45-μm filters twice and 0.22-μm filters once. Buffer volumes included 1.73 ml of SM buffer for adult insect homogenizations and 0.2 ml of TM buffer for pellet resuspension. After ultracentrifugation, the viral pellet was resuspended in a final volume of 50 μl of TM buffer. Particles were negatively stained for visualization using a Zeiss 10C A transmission electron microscope (80 kv). For preparation of infected tissue thin sections, testes of late pupal stage males were dissected in 1× PBS + 0.1% Tween. Tissue was immediately fixed in 2% glutaraldehyde in 0.2M phosphate buffer (pH 7.4) for 2 h at room temperature, washed in 1× PBS three times, and postfixed with 1% osmium tetroxide in buffer for 1 h on ice. Following three 10-min washes in buffer, tissue was dehydrated in graded ethanol series (50% to 100%) and propylene oxide and then embedded in Epon/Araldite resin. Random thin sections of approximately 70 nm were cut using a diamond knife and ultramicrotome (Reichert-Jung), mounted on grids, and stained with 2% uranyl acetate and Reynolds lead citrate for 20 and 15 min, respectively.

## Abbreviations

CI: cytoplasmic incompatibility
RT-qPCR: real-time quantitative polymerase chain reaction
TEM: transmission electron microscopy
